# Supplementary material for: Learning curve and surgical outcome of robotic assisted colorectal surgery with ERAS program
Source: Sci Rep. 2022 Nov 29;12:20566. doi: 10.1038/s41598-022-24665-w (PMC9709162; doi:10.1038/s41598-022-24665-w)
Supplement: Supplementary file 2 — Supplementary Information 2. [file 41598_2022_24665_MOESM2_ESM.zip › RAL Raw data-Ñ[▒K/114/OP2_ñw╝╨░O▒Kñσ.pdf]

正式摘要(電子簽章：000000225809439)

請選擇...reporter

臺中榮民總醫院

Operation Note

[redacted]

- 手術日期：2021/09/25
- 手術醫師：劉嘯天，賴家鈺，CR 蔡易儒
- Anesthesia：GE
- Position：Supine
- Aseptic agents：Soap，Betadine Alcohol

Diagnosis

- Pre-operative diagnosis : Liver mets, S2, S4, S7,
- Post-operative diagnosis : Ditto.

Surgical Indication

- - Synchronous MRCR of liver.
  - Metastasectomy.
  - ICGR15 = 3.19%; T1/2 = 3.0 mins.

Operation

- - Liver S2 wedge resection.
  - Liver S4 subsegment hepatectomy.
  - Liver S7 subsegment hepatectomy.
  - Cholecystectomy.

Finding

- - Finding:
    = No liver cirrhosis.
    = Tumors within liver S2, S4, S7, surface exposure.
  - Method:
    = Pringle 7 times: 16min21sec, 13min05sec, 07min01sec, 21min42sec, 15min18sec, 15min19sec, 19min28sec.
    = Hepatectomy time between 16:08 and 18:26.
  - Specimen:
    = Total weight 100gm.
    = Liver S2, tumor 1x1cm.
    = Liver S4, two tumors, 5x5cm, 2x1cm,
    = Liver S7, tumor 2x1.5cm.
    = All surgical margin involved.
  - EBL: total 450ml.
  - Transfusion: pRBC 2U, FFP 4U.

Procedure

- Under general anesthesia, patient was put into supine position with lower limbs abduction. CRS performed surgeyr first. GS took over for further liver metastasectomy. Additional Six working poles were made (CRSarranged 5 working poles). Liver was moblized by dissection of right triangular and falciform ligament. Tumor was identified by sonography. Inflow control was performed by intermitten Pringle with extracoporeal tournique. Liver panrechyma transection was performed by CUSA and Harmonica. Tumors were resected en-bloc. Hemovac wwas placed over Morrison's pouch. CRS took over for other surgery.
- Estimated blood loss: 450 c.c
- 器械數量: 正確
- 紗布數量: 正確

Image

- PAINT

- abdomen

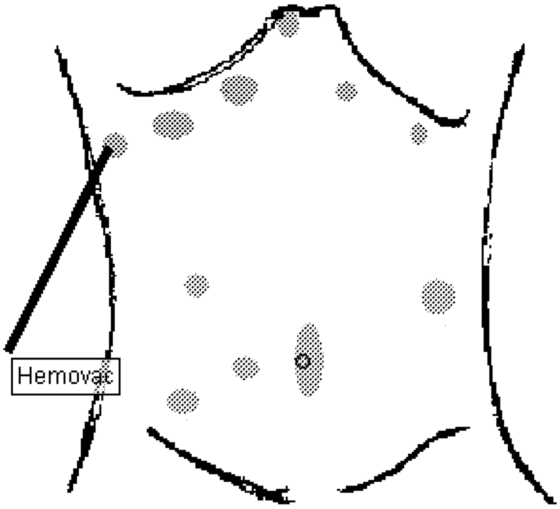

- 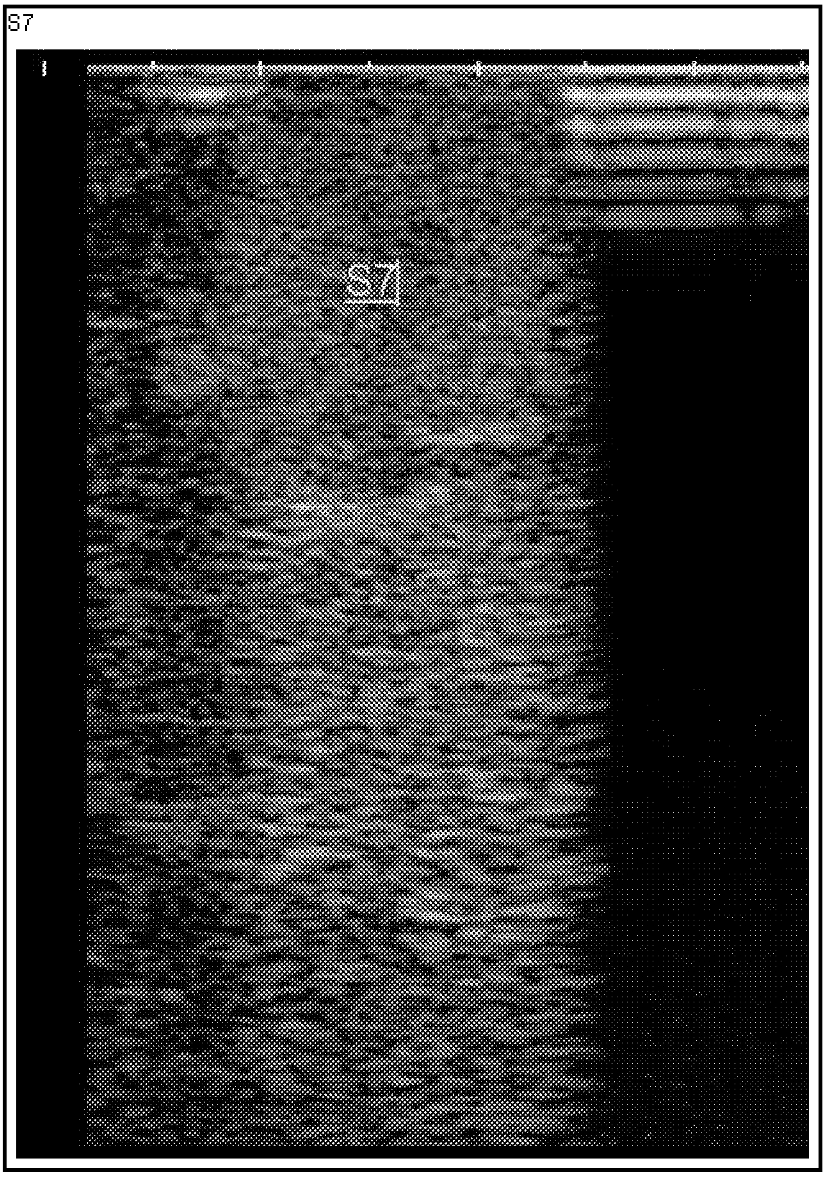

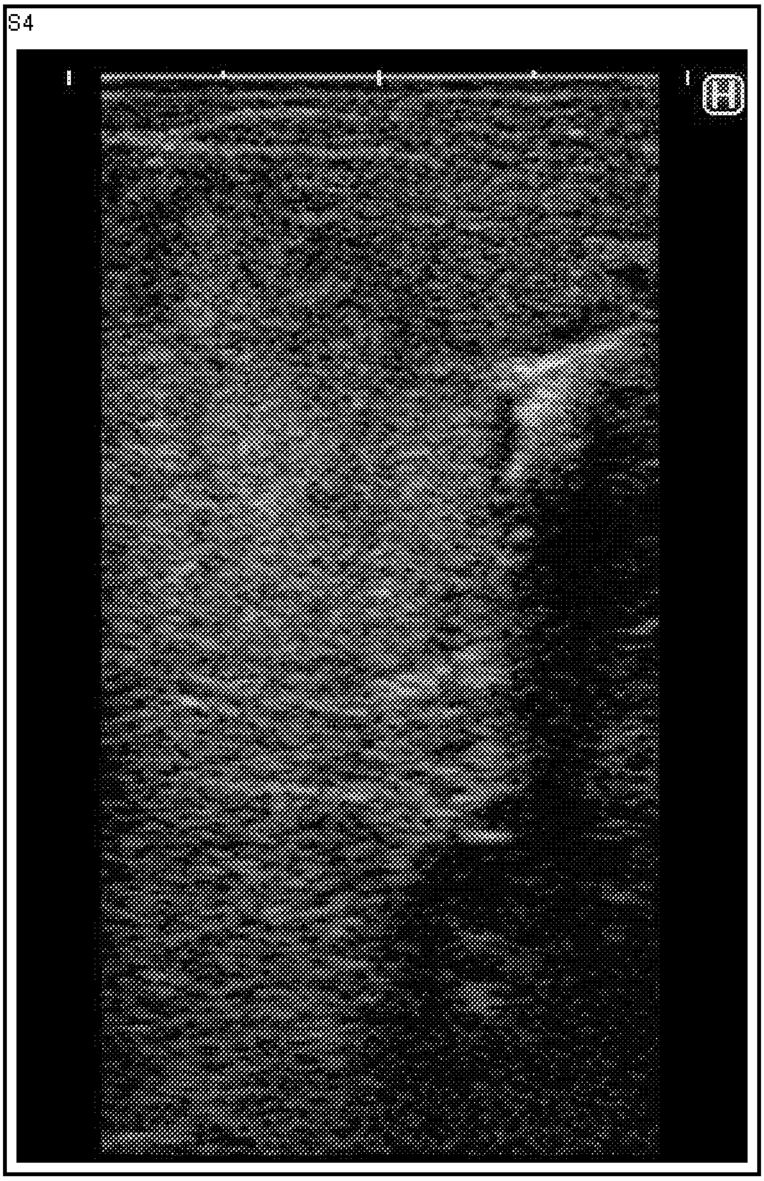
S4

S7

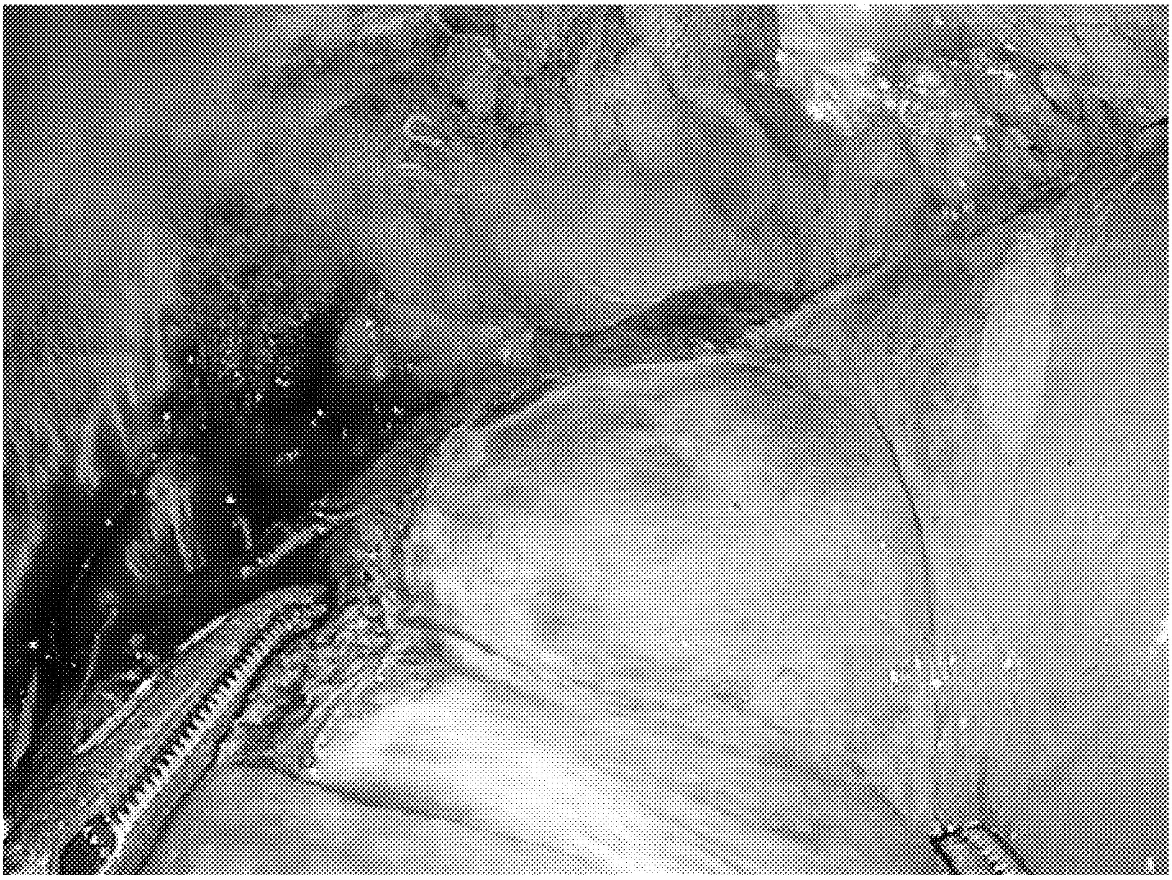

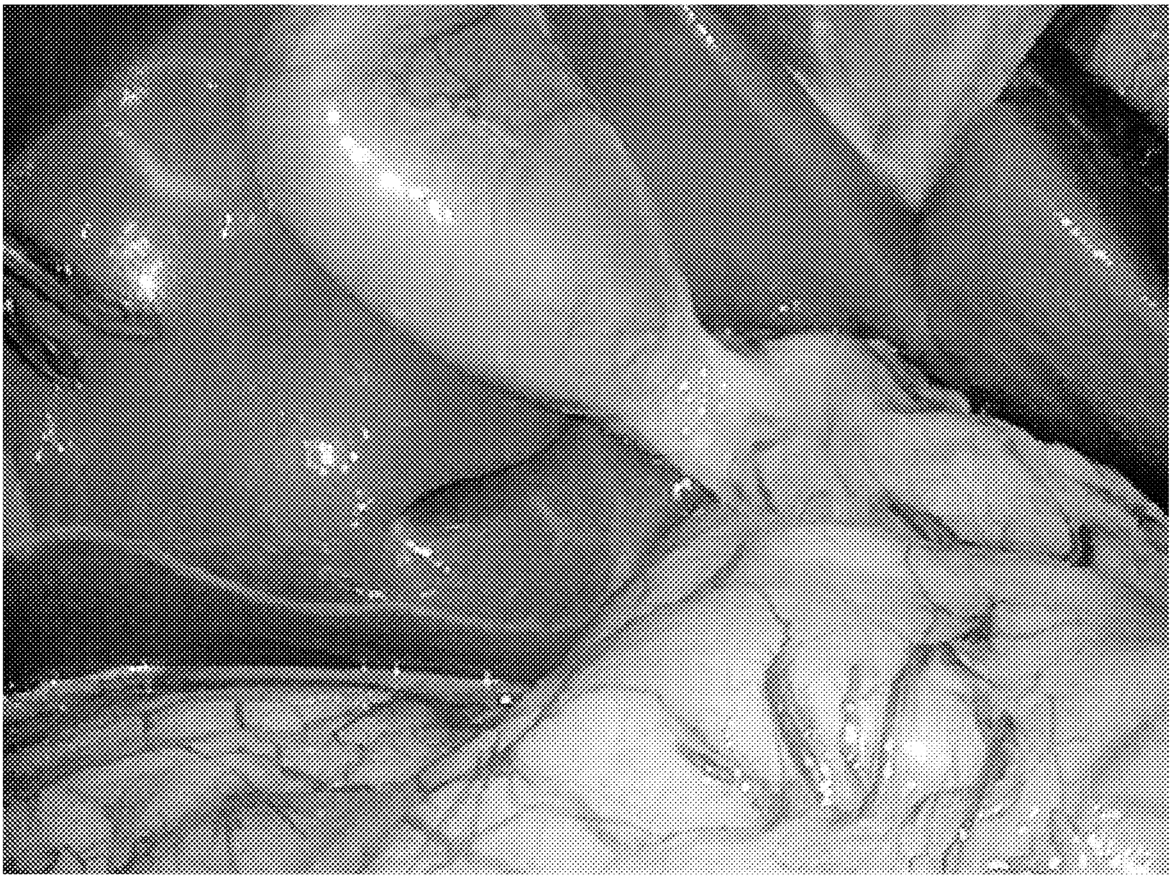

S4

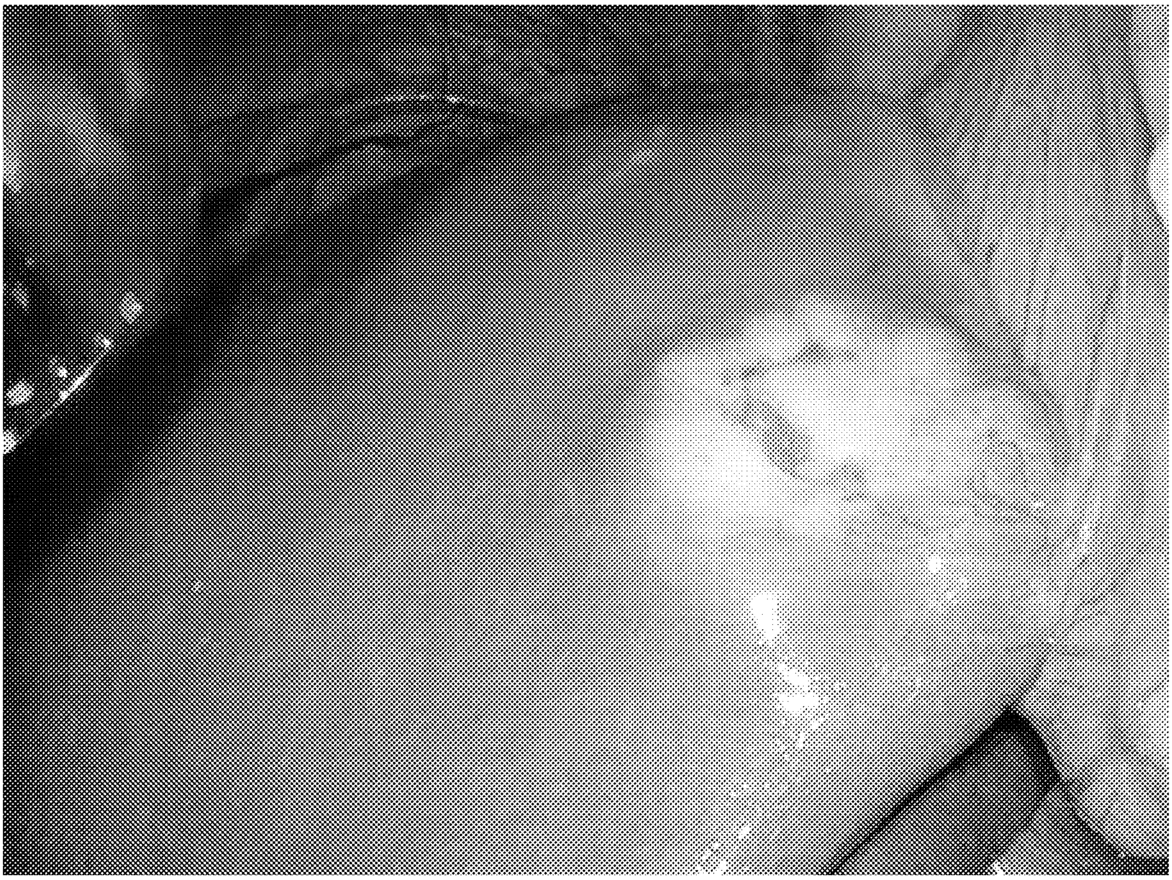

S2

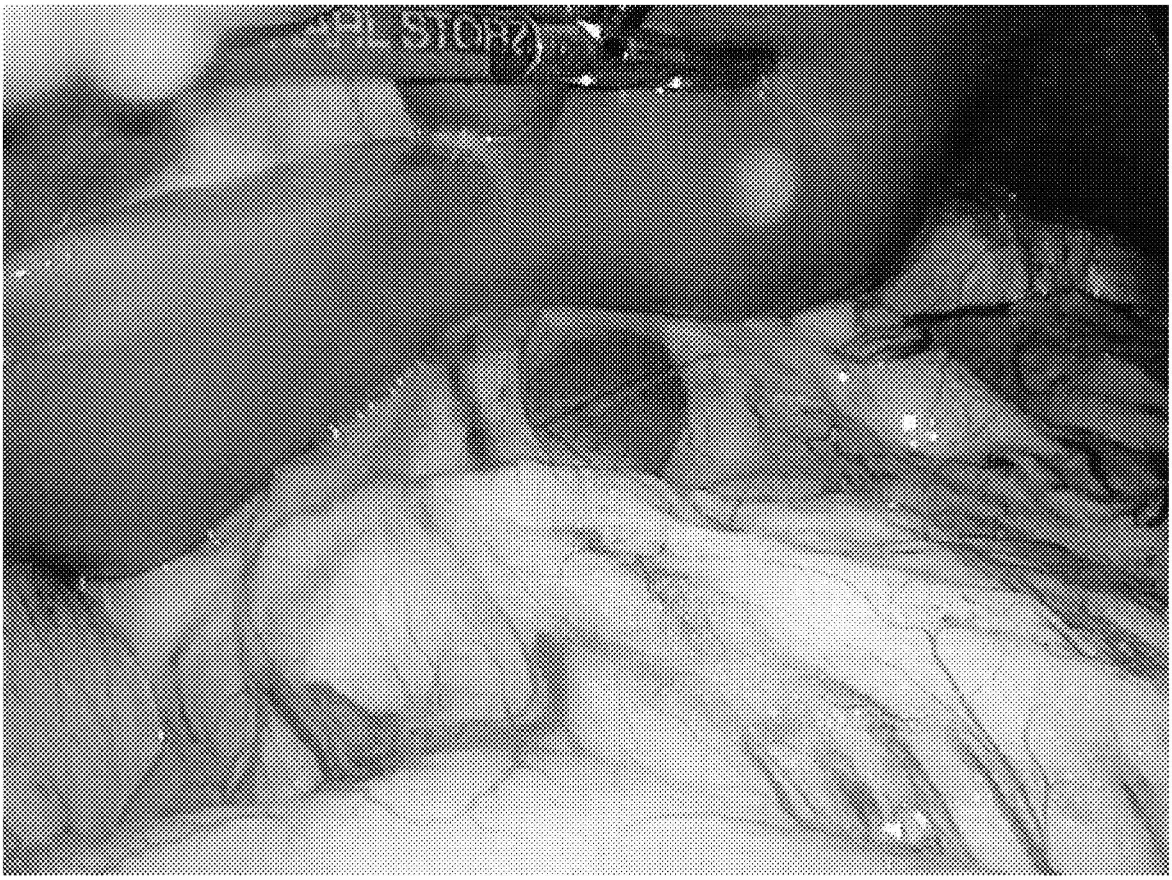

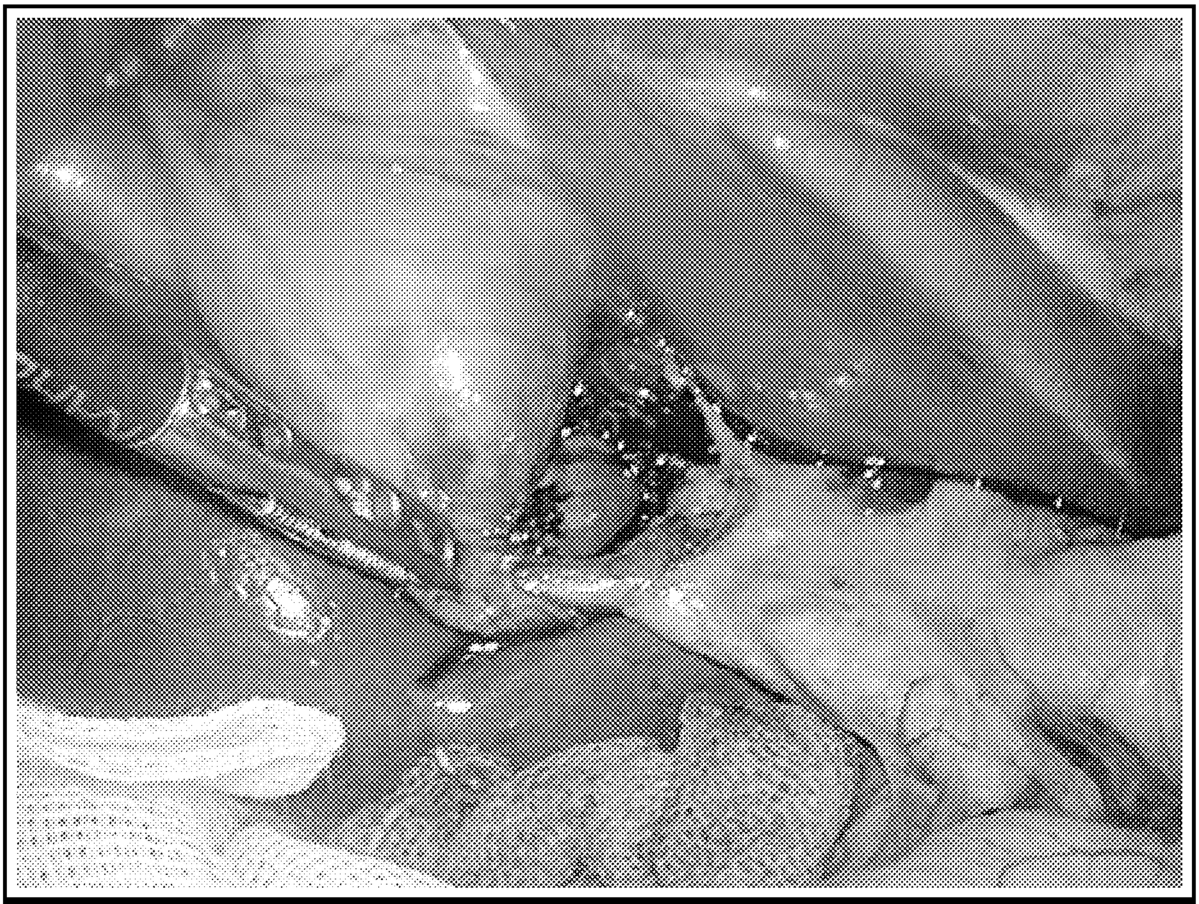

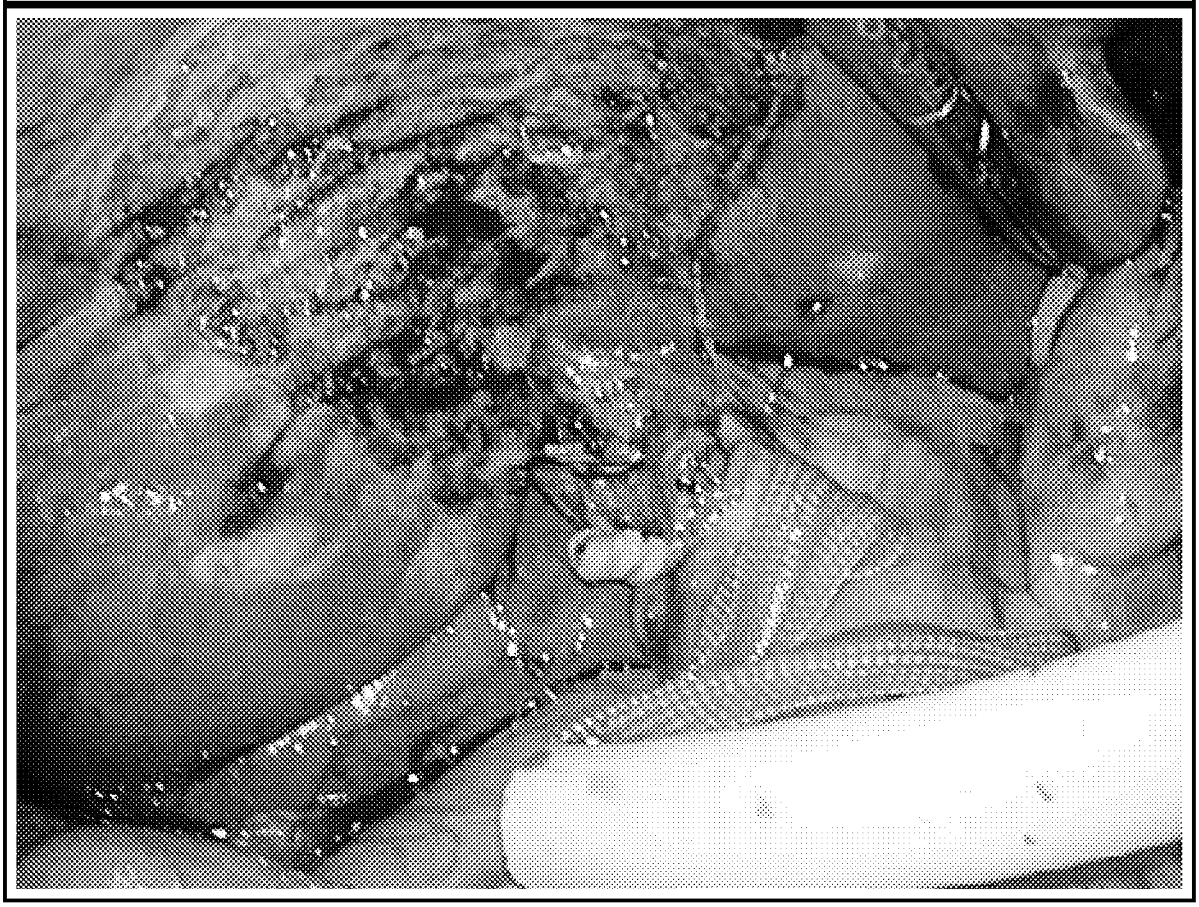

S2

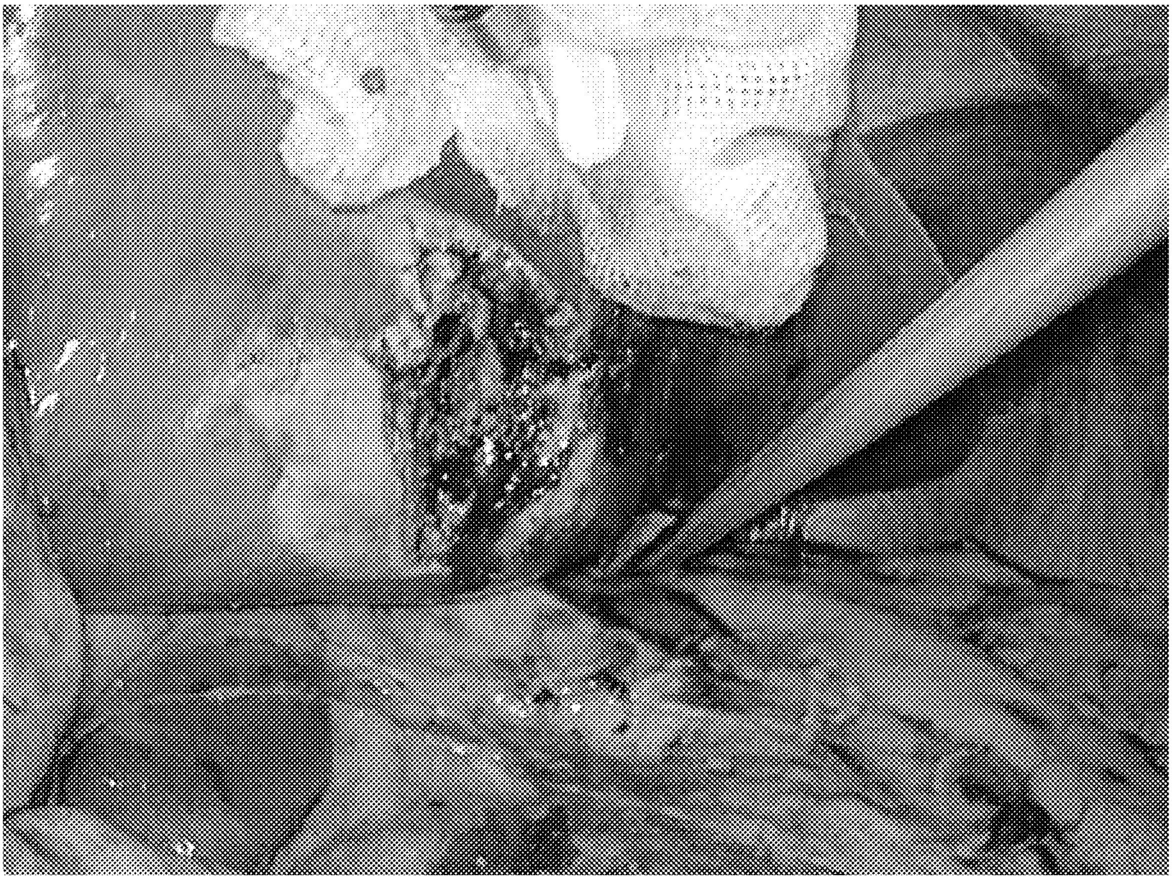

S4

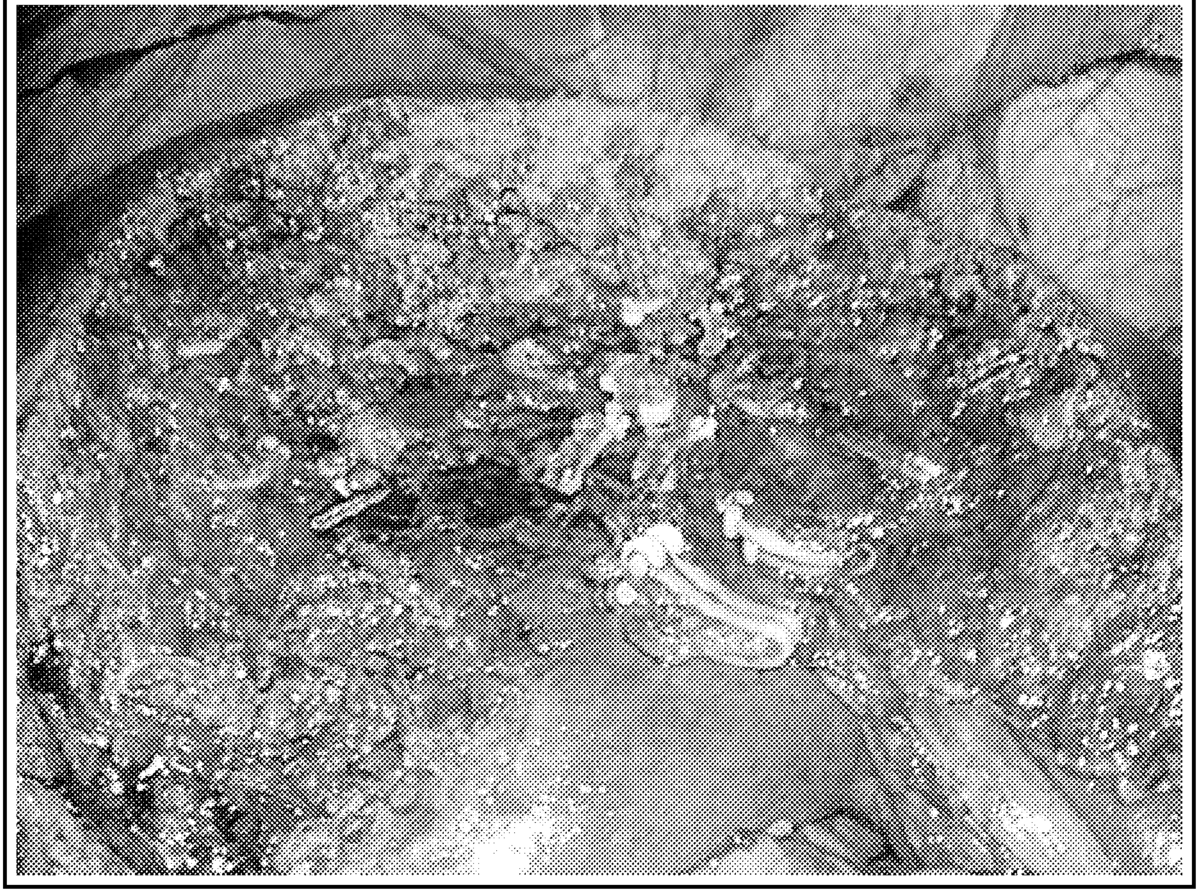

S7

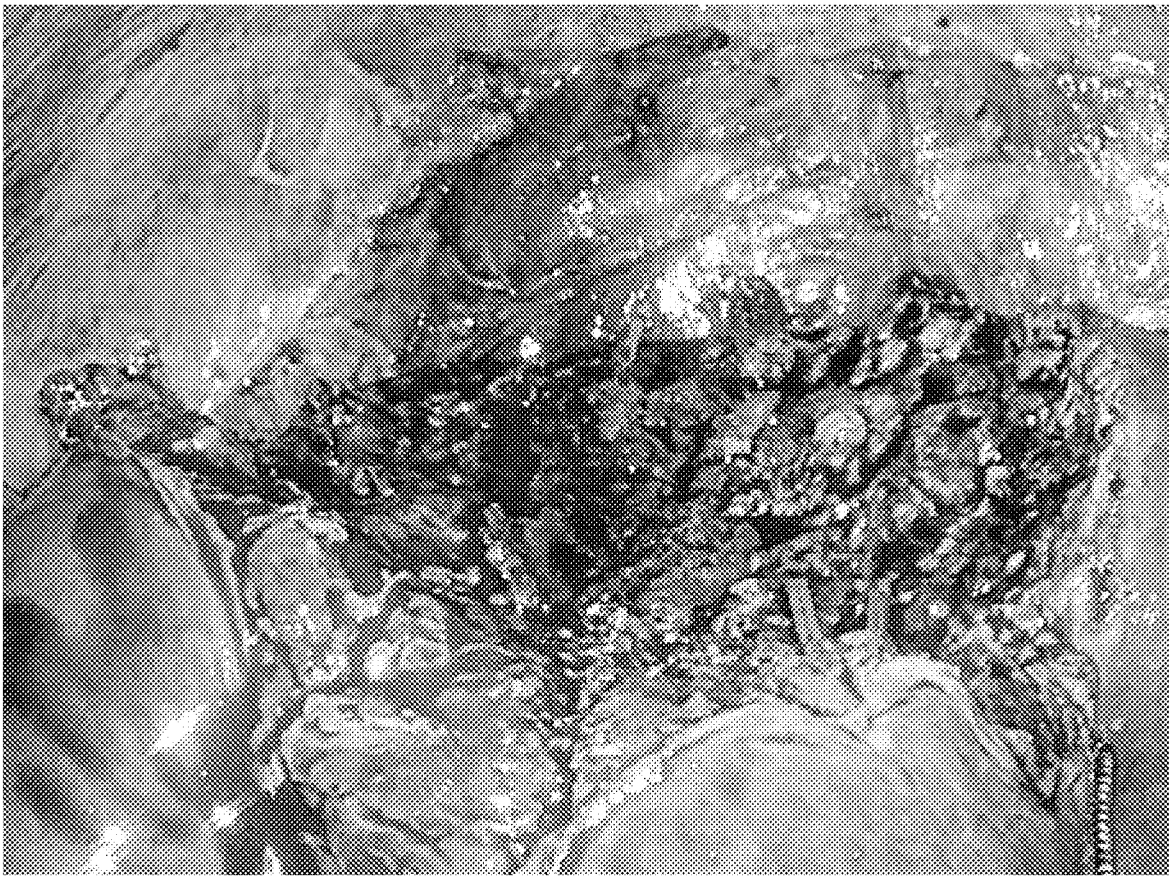

s/p TISSEEL

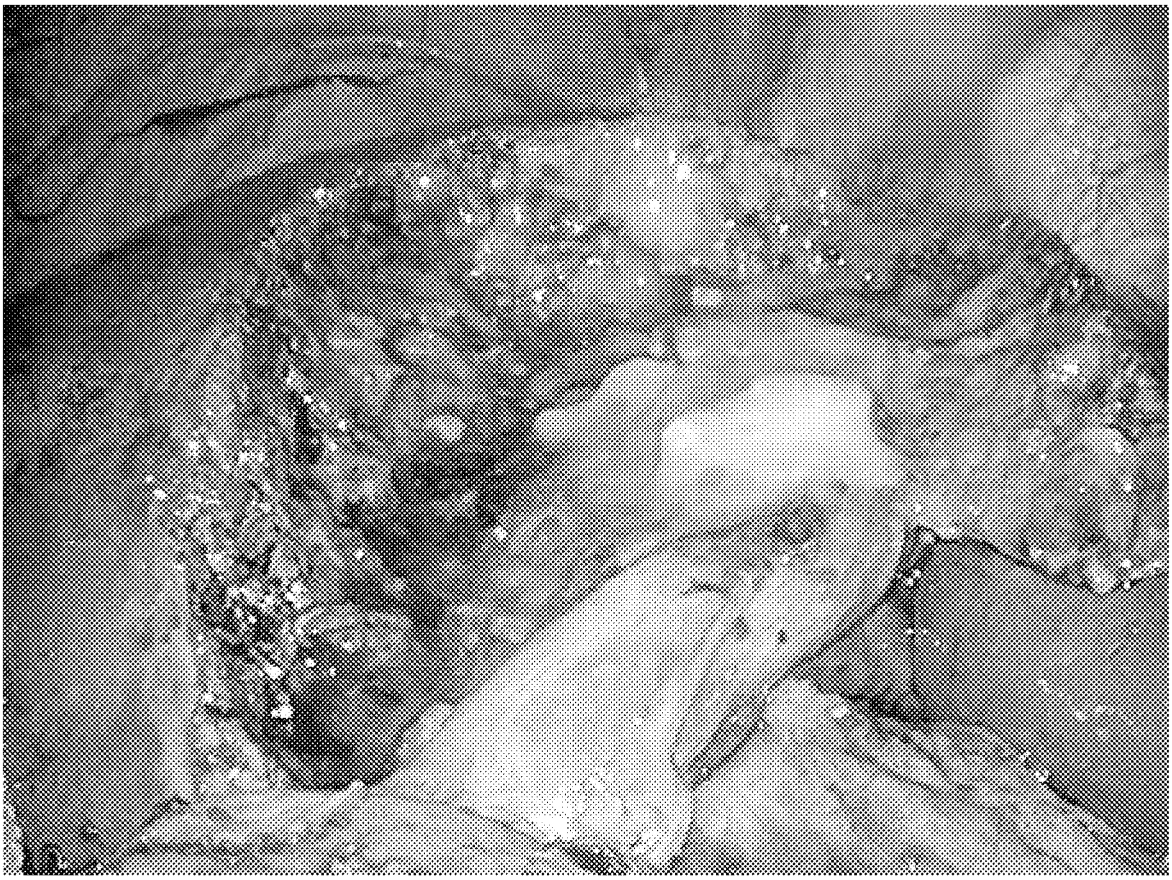

S2

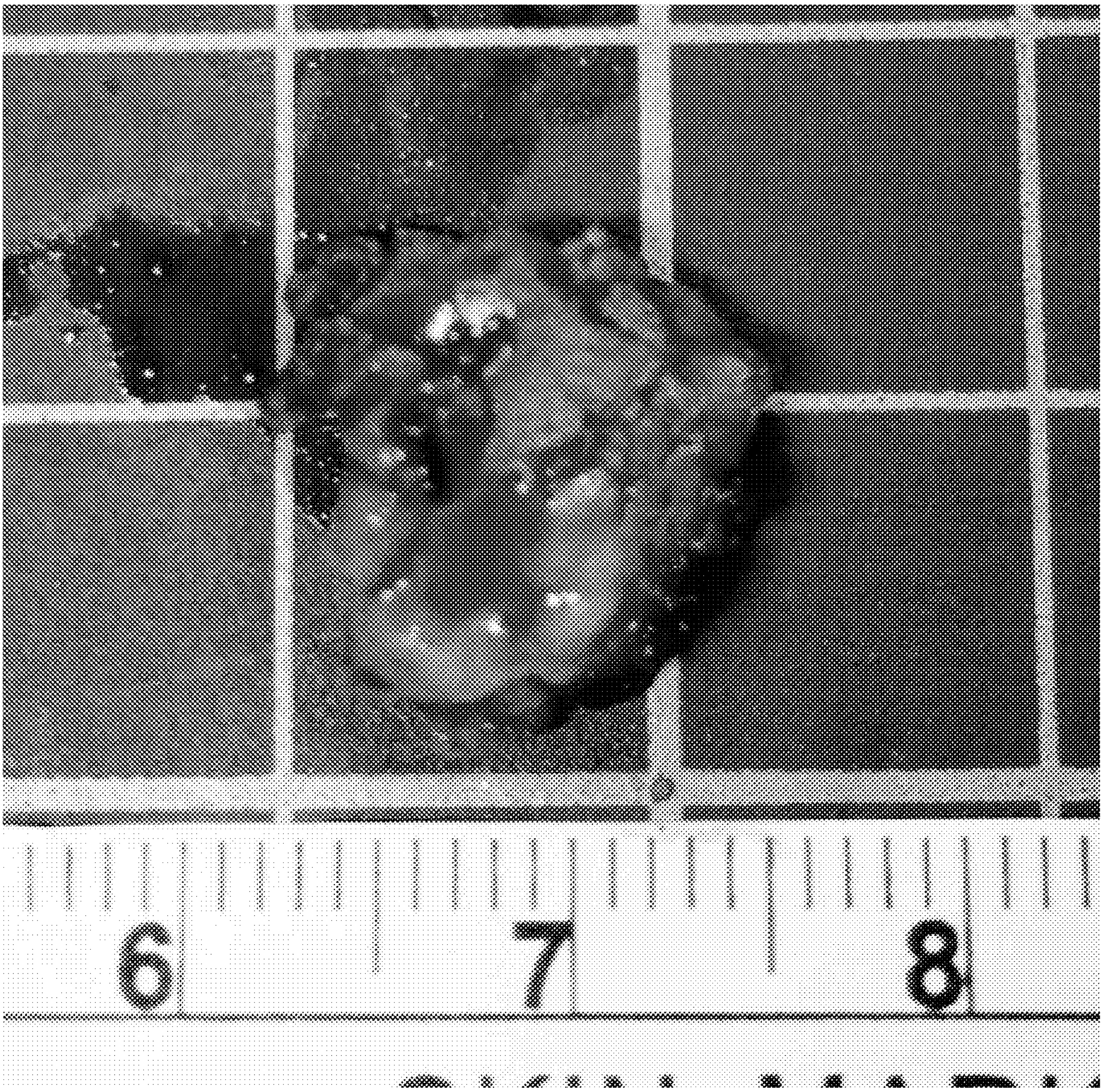

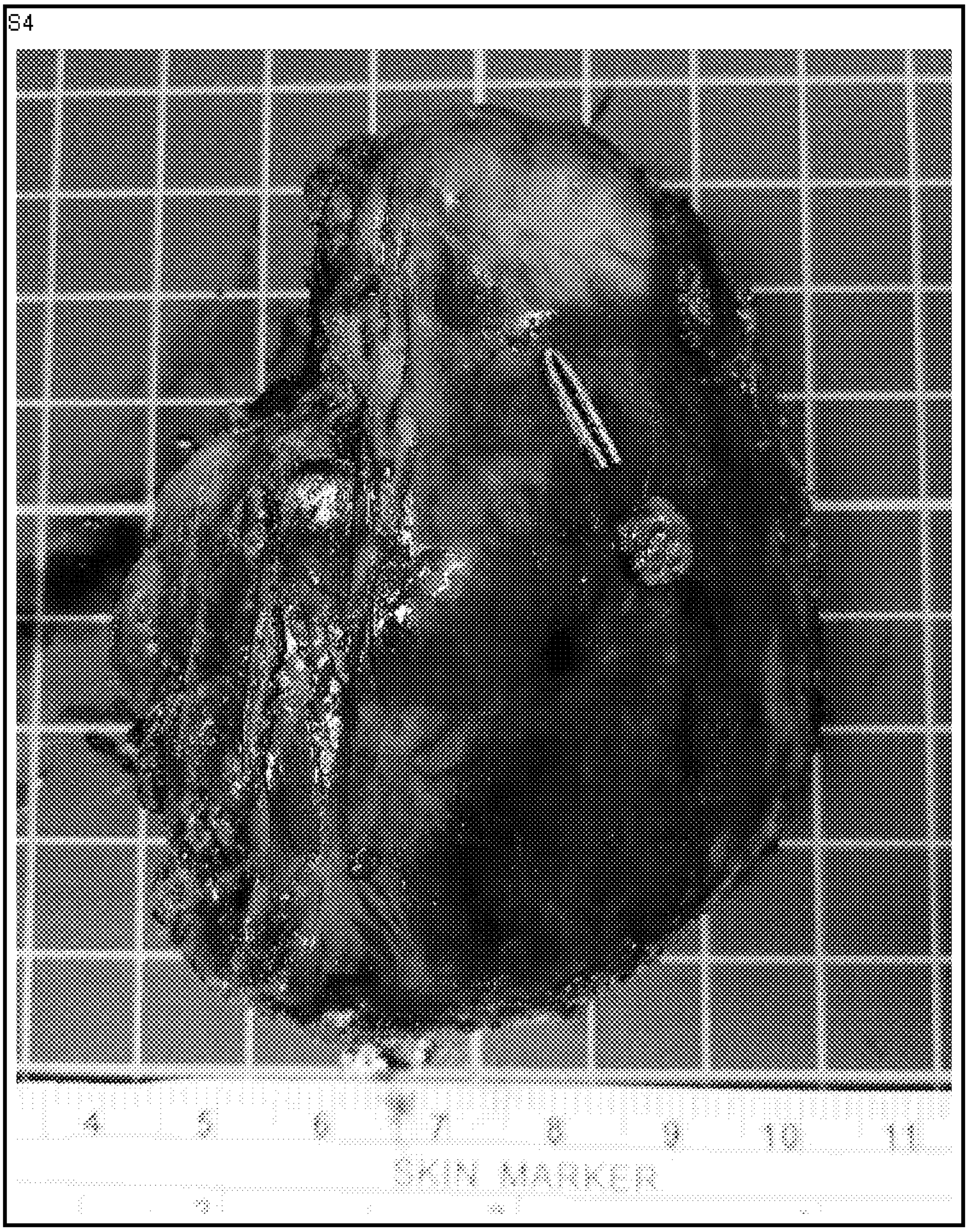
S4
4 5 6 7 8 9 10 11
SKIN MARKER

S4

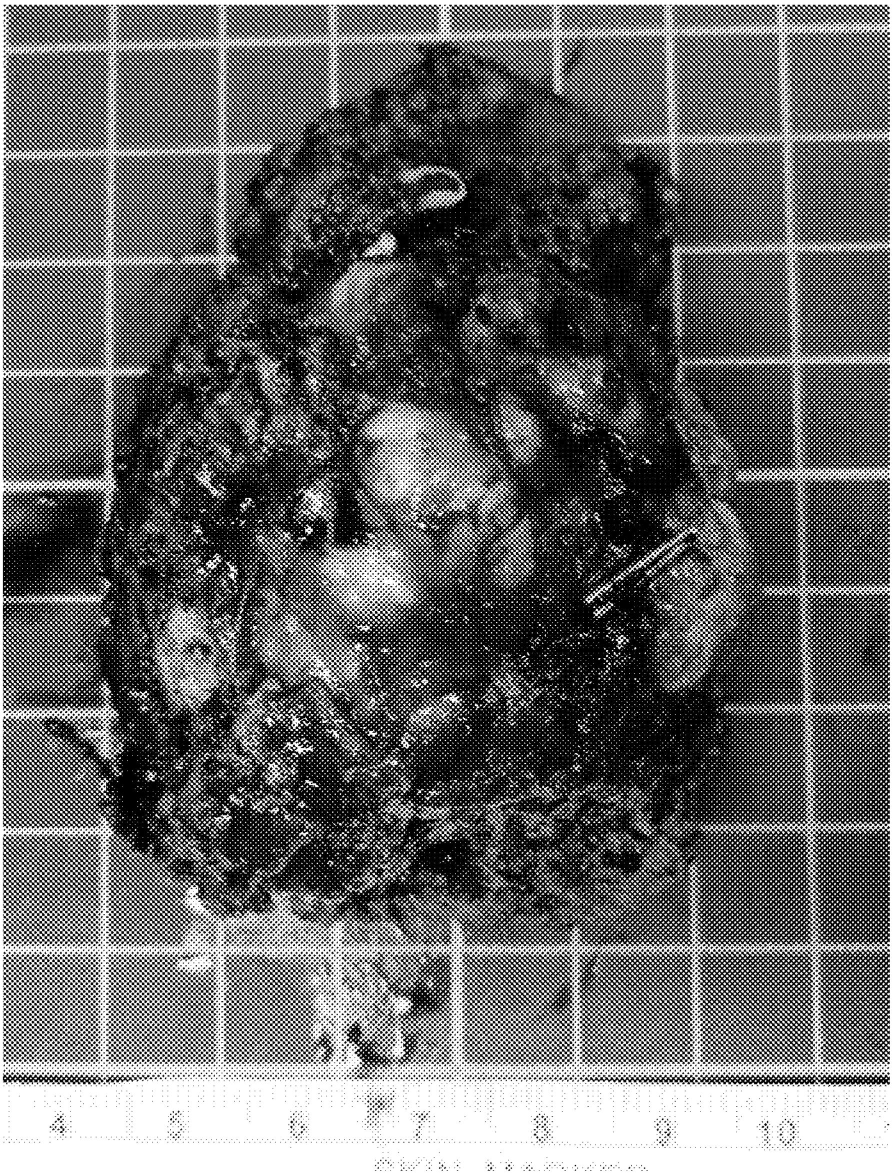

S4

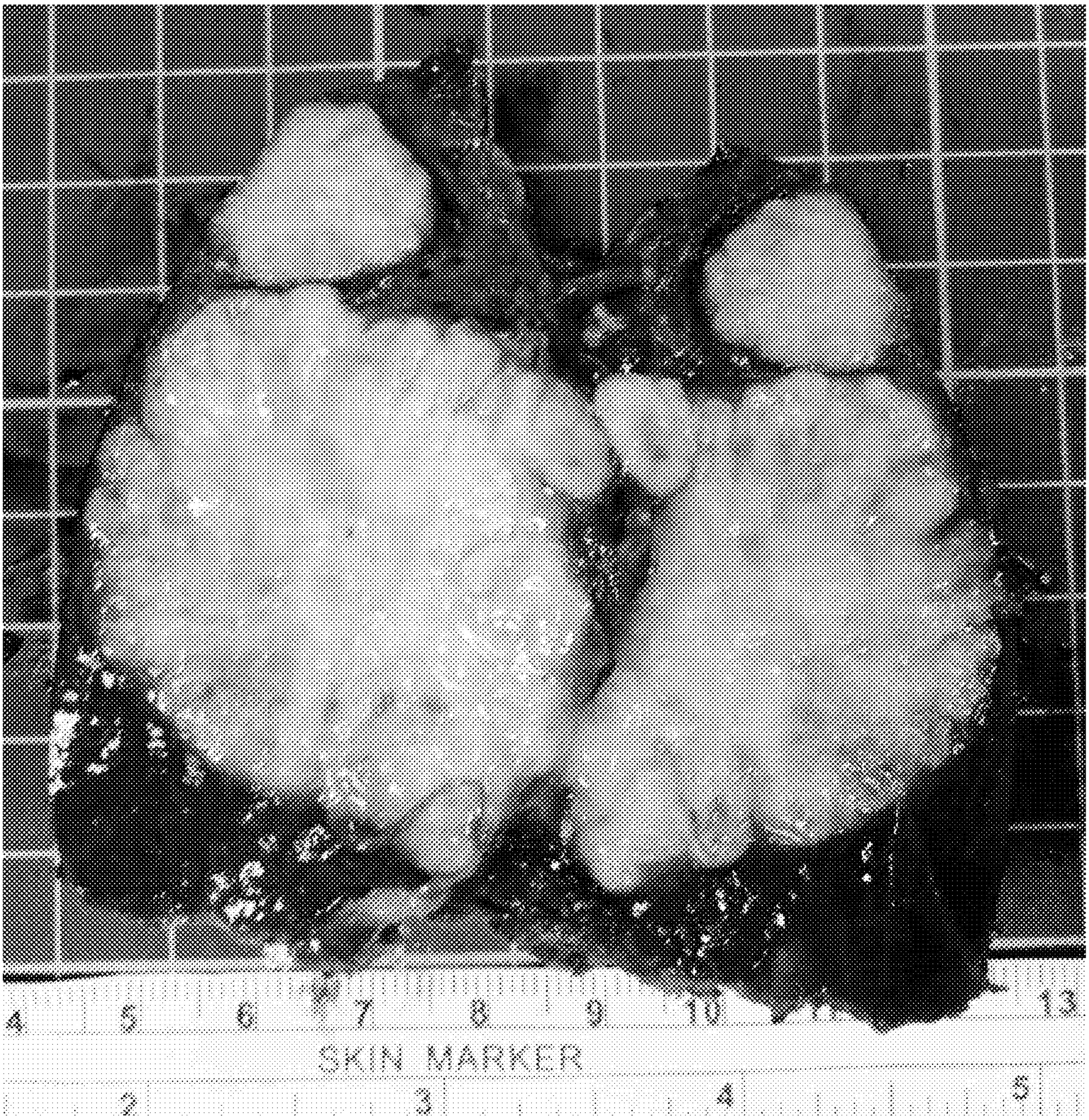

S7

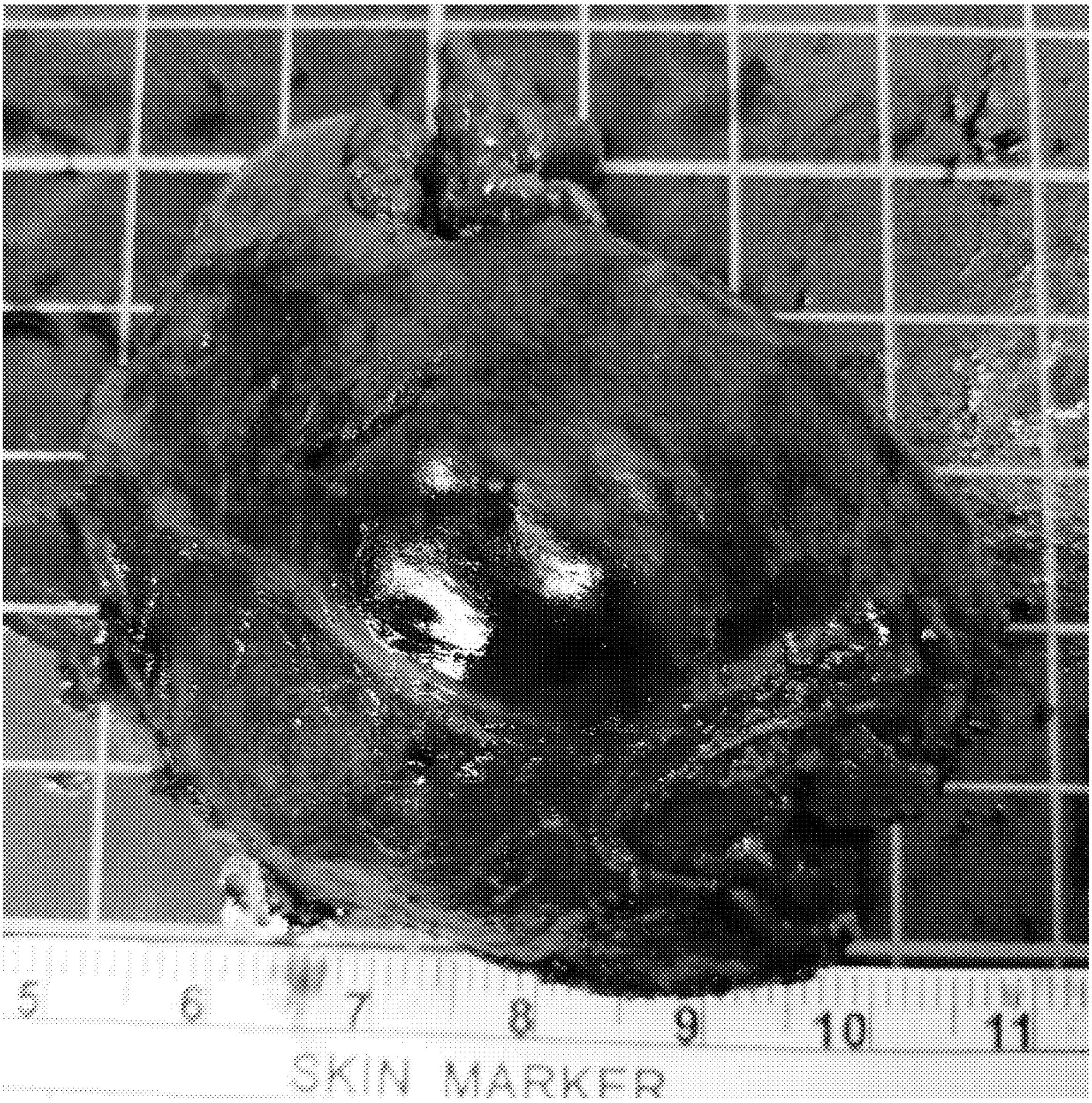

S7

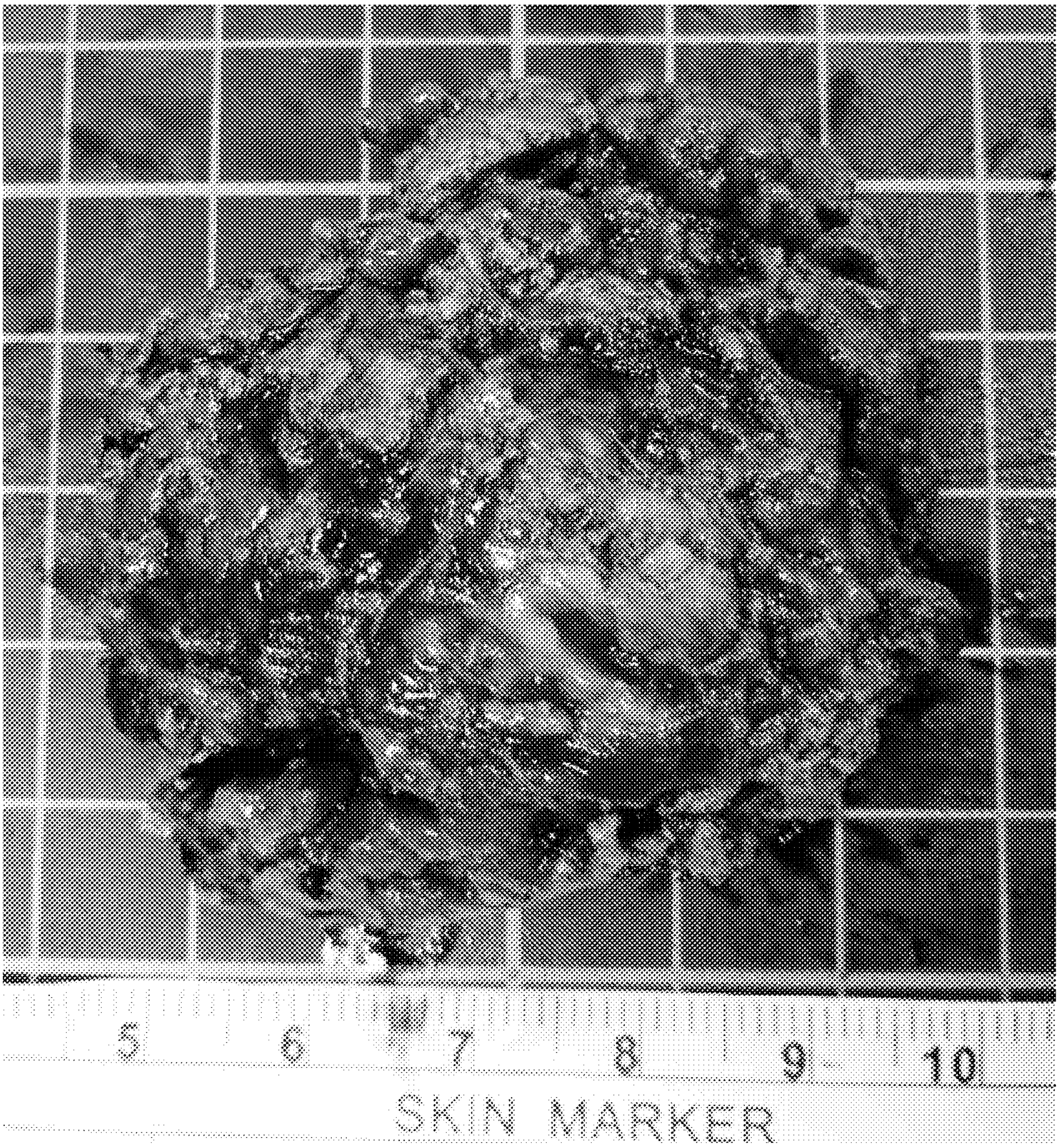

S7

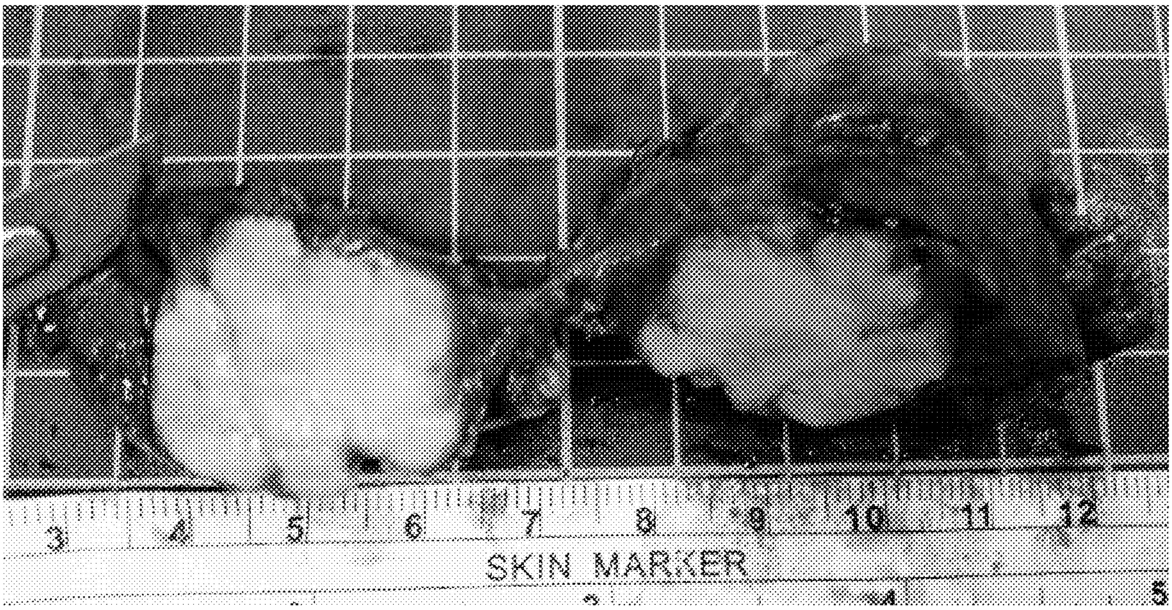

編輯主治 2178L劉嘯天

正式摘要(電子簽章：**000000225809439)**

請選擇...reporter

doctorRank:B
